# Clinical Course and Outcomes of Severe Covid-19: A National Scale Study

**DOI:** 10.3390/jcm9072282

**Published:** 2020-07-18

**Authors:** Moran Amit, Alex Sorkin, Jacob Chen, Barak Cohen, Dana Karol, Avishai M Tsur, Shaul Lev, Tal Rozenblat, Ayana Dvir, Geva Landau, Lidar Fridrich, Elon Glassberg, Shani Kesari, Sigal Sviri, Ram Gelman, Asaf Miller, Danny Epstein, Ronny Ben-Avi, Moshe Matan, Daniel J. Jakobson, Tarif Bader, David Dahan, Daniel A. King, Anat Ben-Ari, Arie Soroksky, Alon Bar, Noam Fink, Pierre Singer, Avi Benov

**Affiliations:** 1 Department of Head and Neck Surgery, The University of Texas MD Anderson Cancer Center, Houston, TX 77030, USA; 2 Israel Defense Forces, Medical Corps, Tel Hasomer, Ramat Gan 01215, Israel; sorkin.alex@gmail.com (A.S.); jacopo669@gmail.com (J.C.); avishaitsur@gmail.com (A.M.T.); gevalandau@gmail.com (G.L.); elon.glassberg@gmail.com (E.G.); kesari.shani@gmail.com (S.K.); ram.gelman@gmail.com (R.G.); danyep@gmail.com (D.E.); drtarifb@gmail.com (T.B.); noamfink@gmail.com (N.F.); avi.benov@gmail.com (A.B.); 3 Department of Plastic and Reconstructive Surgery, Shamir Medical Centre, Zrifin 7033001, Israel; 4 Medical Directorate, Ministry of Health, Jerusalem 9137001, Israel; 5 Division of Anesthesia, Intensive Care, and Pain Management, Tel-Aviv Medical Center, Tel-Aviv University, Tel-Aviv 6423906, Israel; barakc@tlvmc.gov.il (B.C.); danaka8@gmail.com (D.K.); 6 Outcomes Research Consortium, Cleveland Clinic, Cleveland, OH 44195, USA; 7 Department of Medicine ‘B’, Sheba Medical Center, Tel Hashomer, Ramat Gan 52621, Israel; 8 Sackler Faculty of Medicine, Tel-Aviv University, Tel Aviv 69978, Israel; Shaul.lev124@gmail.com (S.L.); tal.hakim@gmail.com (T.R.); soroksky@gmail.com (A.S.); psinger@clalit.org.il (P.S.); 9 Intensive Care, Hasharon Hospital, Rabin Medical Center, Petach Tikva 49372, Israel; 10 Department of Otolaryngology, Head and Neck Surgery, Rabin Medical Center–Beilinson Hospital, Petach Tikva 49372, Israel; 11 Intensive Care Unit, Shamir Medical Centre, Zrifin 7033001, Israel; ayanadvir@gmail.com; 12 Department of Physiology and Pharmacology, Sackler School of Medicine, Tel Aviv University, Tel Aviv 69978, Israel; fridrich.lidar@gmail.com; 13 The Uniformed Services University of the Health Sciences, Bethesda, MD 20814, USA; 14 The Azrieli Faculty of Medicine, Bar-Ilan University, Safed 5290002, Israel; mmatan@poria.health.gov.il; 15 Department of Emergency Medicine, Assuta Ashdod University Hospital, Ashdod 7747629, Israel; 16 Medical Intensive Care Unit, Hadassah-Hebrew University Medical Center Jerusalem 91120, Israel; Sigals@hadassah.org.il; 17 Department of Medicine, Hebrew University-Hadassah Medical Center, Jerusalem 91120, Israel; 18 Medical Intensive Care Unit, Rambam Health Care Campus, Haifa 31096, Israel; asafmiller@gmail.com; 19 Department of Internal Medicine “B”, Rambam Health Care Campus, Haifa 31096, Israel; 20 Thoracic Surgery Division, Poriya Medical Center, Tiberias 15208, Israel; Rbenavi@pmc.gov.il; 21 Covid-19 Intensive Care Unit, Poriya Medical Center, Tiberias 15208, Israel; 22 Intensive Care Unit, Barzilai University Medical Center, Ashkelon 78278, Israel; danielj@bmc.gov.il; 23 Faculty of Health Sciences, Ben-Gurion University of the Negev, Beer-Sheba 84101, Israel; 24 Department of Military Medicine, Hebrew University, Jerusalem 91120, Israel; 25 Respiratory & Medical (RM) Intensive Care Unit, Meir Medical Center, Kfar Saba 44821, Israel; david.dahan@clalit.org.il (D.D.); danny.a.king@gmail.com (D.A.K.); 26 Pulmonary Department, Meir Medical Center, Kfar Saba 44821, Israel; 27 Meir Medical Center, Kfar Sava 44821, Israel; anatiba@gmail.com; 28 Intensive Care Department, Wolfson MC, Holon 58100, Israel; 29 Department of General Surgery, Kaplan Medical Center, Rehovot 76100, Israel; Traumalon@walla.com; 30 The Hebrew University of Jerusalem, Jerusalem 91120, Israel; 31 Department of General Intensive Care and Institute for Nutrition Research, Rabin Medical Center, Beilinson Hospital, Petach Tikva 49100, Israel

**Keywords:** covid-19, ICU, ARDS, comorbidities, prognosis, mortality

## Abstract

Knowledge of the outcomes of critically ill patients is crucial for health and government officials who are planning how to address local outbreaks. The factors associated with outcomes of critically ill patients with coronavirus disease 2019 (Covid-19) who required treatment in an intensive care unit (ICU) are yet to be determined. Methods: This was a retrospective registry-based case series of patients with laboratory-confirmed SARS-CoV-2 who were referred for ICU admission and treated in the ICUs of the 13 participating centers in Israel between 5 March and 27 April 2020. Demographic and clinical data including clinical management were collected and subjected to a multivariable analysis; primary outcome was mortality. Results: This study included 156 patients (median age = 72 years (range = 22–97 years)); 69% (108 of 156) were male. Eighty-nine percent (139 of 156) of patients had at least one comorbidity. One hundred three patients (66%) required invasive mechanical ventilation. As of 8 May 2020, the median length of stay in the ICU was 10 days (range = 0–37 days). The overall mortality rate was 56%; a multivariable regression model revealed that increasing age (OR = 1.08 for each year of age, 95%CI = 1.03–1.13), the presence of sepsis (OR = 1.08 for each year of age, 95%CI = 1.03–1.13), and a shorter ICU stay(OR = 0.90 for each day, 95% CI = 0.84–0.96) were independent prognostic factors. Conclusions: In our case series, we found lower mortality rates than those in exhausted health systems. The results of our multivariable model suggest that further evaluation is needed of antiviral and antibacterial agents in the treatment of sepsis and secondary infection.

## 1. Introduction

Coronavirus disease 2019 (Covid-19) is caused by severe acute respiratory syndrome coronavirus 2 (SARS-CoV-2). This novel coronavirus was identified as the cause of a pandemic that originated in Wuhan, China, in December 2019. As of 8 May 2020, more than 3.9 million people had been diagnosed with the disease in over 187 countries, with more than 272,000 deaths. [1] The magnitude of this pandemic has overwhelmed health care systems worldwide. Hospitals have been overcrowded with Covid-19 patients, and medical providers have been challenged by shortages of Intensive Care Unit (ICU) beds, ventilators, and essential medical personnel [2,3,4]. Differences in resource availability among countries, an absence of data on Covid-19’s clinical course, and the rapid development of the spread of the pandemic, with insufficient follow-up time, have not allowed informed and balanced decision making. For instance, accurate data on clinical outcomes (death versus discharge or transfer from ICU) of ventilated patients would allow better planning of ventilator distribution and use. In addition, data are lacking on the effectiveness of novel therapies, such as antivirals, and existing therapies, such as glucocorticoids and antibiotics.

As of 8 May 2020, Israel had 16,409 Covid-19 cases, with 245 deaths. Israel has designated over 2600 ICU beds for severely ill Covid-19 patients. However, the country has had no more than 181 severely ill patients at a time [5]. Analyzing data from a non-overwhelmed health system of the developed world may shed light on the “natural history” of this disease. In addition, focusing on patients with established outcomes provides a better understanding of the role of different interventions. In this report, we comprehensively assessed the clinical characteristics, interventions, and outcomes of severely ill patients who were treated in Israel. We also identified factors that were associated with mortality.

## 2. Methods

This retrospective registry study was performed at the Medical Corps-Israel Defense Forces, Israel, which is the national coordinating center for the Israel Covid-19 ICU registry. We enrolled all consecutive patients with laboratory-confirmed SARS-CoV-2 infection who were admitted to one of the ICUs among 13 participating hospitals between 5 March and 27 April 2020. Because of the crisis status declared in Israel and the nature of this retrospective chart review, with minimal-risk research using data collected for routine clinical practice, the Israel Ministry of Health (Jerusalem, Israel) waived the need for an individual institutional ethics board and the need for informed consent from individual patients.

According to the World Health Organization (WHO) guidelines [6], laboratory confirmation of SARS-Cov-2 was defined as a positive result on a real-time reverse transcriptase–polymerase chain reaction assay of nasal and pharyngeal swabs. This guidance was implemented locally with the adjunct of using reverse transcriptase–polymerase chain reaction assay from lower respiratory tract aspirates. After being de-identified, patients’ data were recorded daily on an online questionnaire-based electronic worksheet (SurveyMonkey) that was accessible online to registry associates.

### 2.1. Critical Illness-Defining Conditions

This study included critically ill patients admitted to ICU. Criteria for ICU admission were either acute respiratory distress syndrome (ARDS), sepsis or acute organ failure [7,8]. ARDS was defined as PaO_2_/FiO2a ≤ 300 mmHg with Positive End-Expiratory Pressure (PEEP) or Continuous Positive Airway Pressure (CPAP) ≥ 5 cm H_2_O or non-ventilated; if PaO_2_ was not available, SpO2/FiO2 ≤ 315. Sepsis was defined as life-threatening organ dysfunction caused by a dysregulated host response to a suspected or proven infection and a sepsis-related Sequential Organ Failure Assessment (SOFA) score of ≥2 points. Acute organ dysfunction was defined as respiratory (hypoxemia defined by low PaO_2_/FiO2), coagulation (low platelets), liver (high bilirubin), cardiovascular (hypotension), central nervous system (low level of consciousness defined by Glasgow Coma Scale), or kidneys (low urine output or high creatinine). Patients who died prior to ICU admission and patients without outcome data were excluded.

### 2.2. Data Collection

Clinical data reported in this study were collected within the first 24–120 h following ICU discharge or death. The recorded data included the following: age, sex, medical comorbidities (i.e., smoking status, hypertension, diabetes, ischemic heart disease, chronic heart failure, cancer, chronic kidney disease, immunosuppression, cirrhosis, and dementia), medication history, vital signs, chest X-rays, laboratory studies on admission to the ICU, anti-Covid-19 pharmacological therapy in the ICU (antimalarials, antivirals, anti-inflammatories, and plasma from recovered patients), respiratory support method (invasive or noninvasive mechanical ventilation and oxygen mask), renal replacement therapy, nutrition methods (enteral and total parenteral nutrition), the use of extracorporeal membrane oxygenation (ECMO), complications, and outcome. The number of patients who had died, been discharged, and been transferred to a lower level of care as of 5 May 2020, were recorded; ICU length of stay was also determined.

### 2.3. Statistical Analysis

No statistical sample size calculation was performed a priori, and the sample size was equal to the number of patients treated during the study period. The primary outcome was patient status on discharge from the ICU (i.e., dead vs. alive). Continuous variables are presented as the median and interquartile range (IQR) with 95% confidence intervals (CIs). Categorical variables were expressed as the number of patients (percentage). Differences in the distributions of patient characteristics by median age subgroups and the presence or absence of hypertension were reported using differences with 95% CIs. The distribution of data over the age subgroups was based on the available data for that variable, and the other percentages were calculated using the available data for that subgroup.

The Mann–Whitney rank sum test was used to compare nonparametric continuous variables. χ^2^ or Fisher exact test was used for categorical variables as appropriate. The first step was to study the correlation between death and each covariate via a univariable analysis; this was followed by a preliminary multivariable logistic regression model and a Wald test. Thus, covariates with a univariable *p* < 0.05 were included in a preliminary multivariable Wald regression model. Variables that remained statistically significant (*p* < 0.05) were included in the final multivariable model. All statistical tests were two-tailed, and statistical significance was defined as *p* < 0.05. Analyses were performed using JMP Pro 14.0. The analyses were not adjusted for multiple comparisons, and given the possibility of a type I error, the findings should be interpreted as exploratory and descriptive.

## 3. Results

From 5 March to 27 April 2020, 156 patients with suspected or confirmed Covid-19 were hospitalized at one of the participating centers in Israel. Table 1 shows patients’ demographic and clinical characteristics. Positive SARS-CoV-2 status was confirmed prior to hospitalization in 97 patients (62%); in these patients, the median time from laboratory-confirmed SARS-CoV-2-19 to presentation was 4 days (range = 1 to 18 days). The remaining 59 patients had pending test results for SARS-CoV-2, and their positive SARS-CoV-2 status was confirmed during hospitalization. Patients’ median age was 72 years (IQR = 60–82 years; range = 22–97 years); 64 patients (41%) were aged 76 years and older, and 31 (20%) were younger than 55 years. Overall, 69% (108 of 156) of patients were male, with a similar sex distribution among patients younger than 85 years. Among patients older than 85 years, 66% (19 of 29) were female. There was no significant variance in age distribution (*p* = 0.775, Leven’s test) between centers; at one center, 95% of patients (20 of 21) were male, resulting in a significant variance in sex distribution between centers (*p* = 0.042, Pearson test).

Eighty-nine percent (139 of 156) of patients had at least one comorbidity (Table 1). Hypertension was the most common, affecting 85 (54%) patients, followed by diabetes (62 patients (40%)) and ischemic heart disease (33 patients (21%)). Only 13 patients (8%) had a history of chronic obstructive pulmonary disease, eight of whom (5%) were treated for cancer and five of whom (3%) had immunosuppression (i.e., as a result of organ transplant or chronic treatment with systemic corticosteroids). Only one (1.5%) patient older than 75 years presented without preexisting comorbidities; and 89% (57 of 64 patients) presented with multiple comorbidities. Body Mass Index (BMI) data were available for 109 patients: 46% (50 of 109) of patients were overweight (i.e., BMI between 25 and 30) and 29% (32 of 109) were obese (i.e., body mass index, BMI > 30). Supplementary Table S1 presents patients’ medication histories.

Table 2 presents patients’ vital signs, chest X-rays, and laboratory findings on admission. Acute organ failure was the most common critical illness-defining condition (103 of 156 patients (66%)), followed by ARDS (69 of 156 (44%)) and sepsis (40 of 156 (26%)) (Table 2). Deterioration in inpatients who were initially not classified as critically ill occurred in 56% of patients (88 of 156); in these patients, the median time to deterioration and ICU admission was 3 days (IQR = 2–6 days, range = 1–20 days). Hydroxychloroquine and chloroquine were the most commonly used anti-Covid-19 pharmacological agents administered in the ICU (124 of 156 (79%)), followed by corticosteroids (22%) and antiviral agents (19%) (Table 3). Of note, fresh plasma from patients who had recovered from Covid-19 was administered in seven patients (5%).

Table 3 presents the ICU interventions and organ replacement therapies used. Among 156 patients who were admitted to the ICU, 103 (66%) required endotracheal intubation and mechanical ventilation, and 39 patients (25%) were treated with noninvasive ventilation. There were no differences in mechanical ventilation rates among different age groups (71% of patients younger than 55 years, 61% of patients older than 75 years, and 69% of patients between 55 and 75 years; *p* = 0.725, likelihood ratio); however, noninvasive ventilation was used more frequently in patients younger than 55 years (16 of 31(52%)) than in patients older than 75 years (6 of 64 (9%)) and patients between 55 and 75 years old (17 of 61 (28%)), *p* < 0.001, likelihood ratio). Tracheostomies were placed in 14% (13 of 103) of ventilated patients. ECMO and renal replacement therapy were used in 4% (6 of 156) and 9% (14 of 156) of patients with acute respiratory and renal failure, respectively. Seventy-four (47%) patients required tube feeding via a nasogastric tube, and 15 (10%) were fed with total parenteral nutrition. A secondary infection was diagnosed in 27 (17%) patients, and 21 (13%) developed sepsis. An acute kidney injury developed in 40 (27%) patients, and an acute cardiac injury developed in eight (5%) (Table 3).

As of 8 May 2020, the median (IQR) length of stay in the ICU was 10 days (IQR = 5–17 days; range = 0–37 days). Among patients who were intubated (*n* = 103), the median (IQR) ventilation time was 13 days (range = 7–19 days) compared to 7 days (range = 2–11 days) in non-intubated patients (*p* < 0.001, Analysis of variance—ANOVA). We also found a significant correlation between patients’ age and length of stay: the median (IQR) length of stay in the ICU was 14 days (6–21 days) in patients younger than 55 years and 4 days (2–11 days) (*p* = 0.004, correlation coefficient = −0.225) in patients older than 85 years. With a total of 87 deaths, the overall mortality rate was 56%; 25 (16%) patients were discharged home, and 44 (28%) experienced clinical improvement and were transferred to a lower level of care (i.e., rehabilitation or Covid-19 internal medicine departments). We found a significant difference in the length of ICU stay between patients who died (median = 7 days; IQR = 3–12 days) and those who experienced improvement and were discharged from the ICU (median = 15 days; IQR = 9–21 days).

A univariate analysis revealed that patients’ age, sex, comorbidity status, sepsis, white blood cell count, antiviral therapy, antimalarial therapy, and length of ICU stay were all statistically significant predictors of outcome (Table 4). We included the significant variables in a multivariable regression model. This analysis revealed that older age (OR = 1.08 for each year of age; 95% CI = 1.03–1.13), the presence of sepsis (OR = 1.08 for each year of age; 95% CI = 1.03–1.13), and short length of ICU stay (OR = 0.90 for each day; 95% CI = 0.84–0.96) were the only independent prognostic factors.

## 4. Discussion

In this multicenter case series, we evaluated 156 critically ill patients who were admitted to ICUs in Israel with laboratory-confirmed SARS-CoV-2 from 10 March to 5 May 2020. While the pandemic has been subsiding in some parts of the world, there is still a stable plateau in the western world and North America. Covid-19 adversely impacts health systems, mostly due to major uncertainties regarding the outcomes of this disease. These uncertainties explain the aggressive responses of policy makers that have detrimentally affected societies and economies around the globe [9].

Here, we focused on critically ill patients because (1) they are at the highest risk, (2) their clinical course and management are poorly defined, and (3) they demand the most resources and care [10]^.^ Most of the recently published data were collected during or near the peak of the outbreak, and careful evaluation revealed that outcome data were available for only a minority of patients and were not available for many hospitalized patients. We focused on survival data in our analysis of critically ill Covid-19 patients. The Israeli health system has not reached its maximal treatment capacity (Supplementary Figure S1), which has allowed us to provide the best possible care, with minimal to no resource constraints for each patient. This unique report provides a clear understanding of the course of the disease at its extreme and sheds light on its clinical course in a non-overwhelmed health system. In Israel, the disease course is unique as the health system was underutilized; and most, if not all patients received best possible care with minimal resource constraints. This, might also explain the relatively low mortality compared to reports from other regions.

The patient population in our cohort was similar to those reported elsewhere in the world. The majority of patients were older men, and a large proportion presented with multiple comorbidities. Most of our patients were admitted with ARDS and respiratory failure and required respiratory support, similar to the patients described in reports from China [3]^.^ Approximately two-thirds of the patients required invasive mechanical ventilation, mostly in older patients. Despite these similarities, we found a difference in outcomes.

The following mortality rates have been found for Covid-19 patients in the ICU for whom outcome data are available (i.e., excluding patients who were still being treated in the ICU at the time of the report): 61% (Lombardy, Italy [2]), 78% (New York City, NY, USA [4]), 79% (Wuhan, China [11]), 57% (Seattle, WA, USA [12]), 85% (Washington state, USA [13]), and 67% (China [3])) The mortality rate in our series was 56% at the time of data cut-off. There are several possible reasons why our rate was lower. First, use of the health system for Covid-19 in Israel never reached its maximal capacity, allowing longer ICU stays. The median ICU length of stay in our cohort was 10 days, with an even longer stay in patients who survived (median, 15 days). In the above-mentioned studies, the median length of ICU stay ranged between 4.1 and 8 days [1,2,12,13]. A longer ICU stay allows patients to be weaned more slowly from the ventilator and allows longer follow-up to monitor response to novel therapies that, in turn, might affect patients’ outcomes and prevent relapses and readmission or subsequent death. Second, the lag between the outbreak in China, Italy, and Spain and Israel allowed Israel’s health system to adjust and implement some of the lessons learned in regions that had been severely impacted by the virus; these included the need for an isolation regimen, personal protective equipment, and “capsules” that allow complete separation between providers who treat SARS-CoV-2-infected patients and those who do not. Lastly, novel and advanced therapies, such as plasma derived from patients who have recovered from Covid-19 and ECMO, were readily available for these patients. ECMO was performed in six patients in our study who were younger (median age = 48 years, IQR = 39–49 years) than the mean of the cohort, and only one patient treated with ECMO died. Although the differences in survival rates between patients who were and were not treated with ECMO were statistically significant, we have refrained from making a conclusive statement about the therapeutic yield of ECMO; however, we recommend its consideration in younger critically ill patients.

By evaluating patient and outcome data, we were able to assess the effects of various patient and disease factors on outcome. While multiple factors were associated with death, our multivariable regression model indicated that only older age, longer length of ICU stay, and the presence of sepsis were independent predictors of outcome. Similar to our data, some studies have reported the presence of comorbidities, such as hypertension, in severely ill patients [2,3,14]. Hypertension and associated therapies (e.g., angiotensin-converting enzyme and angiotensin receptor blockers) have been found to be associated with mortality [15]^.^ The results of our multivariable analysis suggest that these factors (i.e., comorbidities and related medication used) are associated with patients’ age rather than with actual outcome; thus, the mortality rate is also associated with age.

We were able to include novel therapies in our analysis, some of which were considered compassionate (e.g., remdesivir). As a retrospective cohort, we suspect that our study was not designed to evaluate the efficacy of different anti-Covid19 therapies and was underpowered. Our data support the prospective evaluation of antiviral and antimalarial agents in critically ill patients. Our data showed no difference in the outcomes of patients treated with glucocorticoids. However, the results of a recent study suggested that glucocorticoids are associated with better clinical outcomes in patients with Covid-19 and ARDS [16]. On the basis of the results of previous studies that investigated phylogenetically similar viruses (SARS-CoV-1 (2003) and Middle East respiratory syndrome coronavirus (MERS-CoV)), we hypothesize that glucocorticoid treatment was associated with a higher subsequent plasma viral load, a longer viremia duration, and worse clinical outcomes in our cohort [17,18,19,20]. This hypothesis is also in agreement with the better overall outcomes in patients who were treated longer in the ICU.

A large proportion of patients in this series presented shock that required vasopressor support. Many of these patients presented sepsis or developed secondary infection and septic shock. Unlike other reports that have demonstrated no bacterial or viral coinfection, we found the presence of sepsis in some patients; this finding suggests that, similar to seasonal influenza, Covid-19 is associated with bacterial coinfection due to pathogens that colonize the nasopharynx, such as Staphylococcus and Streptococcus, in critically ill patients [21]. This might also explain the lack of efficacy of glucocorticoid treatment that might be hindered. Of note, most of our patients were treated with antibiotics for over 24 hours. We recommend the prospective evaluation of the role of antimicrobial therapy in critically ill patients.

## 5. Limitations

This study has several limitations. First, it was a retrospective study. As such, some variables were not available for assessment. For example, computed tomography scans were not done routinely in all cases, and in the few cases when it was performed, it was done in a single time point. These considerations precluded the utilization of Computed Tomography (CT) scan as a marker for disease severity or prognosis in our cohort. However, the data were collected no longer than 5 days after outcomes were achieved for each patient. Second, although our data are comprehensive and complete, we could not include all of the collected variables in the regression model because of our considerably underpowered number of events. That said, considering the population size in Israel (nearly 9 million citizens), the reported numbers of cases and deaths reflect the relative numbers of events in other countries affected by Covid-19. Third, while the post-discharge follow-up was short, the follow-up time in the hospital was considerably long compared with the course of the disease, and more data were available with regard to the reported mortality data and length of stay data reported in other studies.

## 6. Conclusions

In this nation-based registry study of critically ill patients with Covid-19 who were admitted to ICUs in Israel, the majority of patients were 55 years and older men, and a large proportion required mechanical ventilation. The overall mortality rate was 56%; increasing age, shorter ICU stay (median, 7 days versus 15 days), and the presence of sepsis were independently associated with death. We found no association between coexisting conditions and outcome. Our findings also highlight the importance of novel therapies, antibiotics use, and the availability of resources such as ICU beds and ventilatory support in the treatment of patients with Covid-19. These data will inform quality improvement efforts and counseling of high-risk Covid-19 patients.

## Figures and Tables

**Table 1:** Demographic and clinical characteristics of critically ill patients admitted to the ICU for Covid-19.

Variable (No.)	Total (*n* = 156)	Discharged Alive (*n* = 69)	Died (*n* = 87)	*p* Value
Age, Median (IQR)	72 (60–82)	61 (49–69)	80 (72–87)	<0.001
Sex				0.003
Female (%)	48 (31)	13 (19)	35 (40)	
Male (%)	108 (69)	56 (81)	52 (60)	
**Comorbidities**				
Cancer				
Active	8 (5.1)	3	5	0.6
History	14 (9)	2	12	0.02
Cardiovascular Disease				
Hypertension	85 (54.5)	23	62	<0.00001
Coronary Artery Disease	33 (21.2)	9	24	0.03
Congestive Heart Failure	17 (10.9)	3	14	0.02
Arrhythmia	17 (10.9)	3	14	0.02
Chronic Respiratory Disease				
Chronic Obstructive Pulmonary Disease	13 (8.3)	3	10	0.1
Immunosuppression ^a^	6 (3.8)	2	4	0.5
Kidney Disease ^b^	24 (15.4)	4	20	0.003
Liver Disease	2 (1.3)	1	1	0.8
Obesity	109 (69.9)			
Obesity (BMI < 25)	27 (24.8)	6	21	0.01
Obesity (BMI 25–30)	50 (45.9)	29	21	0.01
Morbid Obesity	32 (29.4)	18	14	0.1
Metabolic Disease				
Diabetes ^c^	62 (39.7)	20	42	0.01
Dyslipidemia	24 (15.4)	16	8	0.02
Hypothyroidism	15 (9.6)	6	9	0.7
Smoker				
Never	143 (91.6)	61	81	0.3
Stopped Over a Year Prior Hospitalization	11 (7.1)	6	5	0.5
Active	2 (1.3)	1	1	0.8
Dementia	21	0	21	<0.00001
Comorbidities				
None	30	24	6	<0.00001
1	27	18	9	0.01
2	30	11	19	0.3
3	30	8	22	0.03
>3	39	8	31	0.0006
Critically Ill Defining Condition *				
Acute Respiratory Distress Syndrome (%)	69 (44)	34 (49)	35 (40)	0.258
Acute Organ Failure (%)	103 (66)	42 (61)	61 (70)	0.226
Sepsis (%)	40 (26)	9 (13)	31 (36)	0.001
Length of ICU stay (days), median (IQR)	10 (5–17)	15 (9–21)	7 (3–12)	<0.001

Abbreviations: Covid-19, coronavirus disease 2019; IQR, interquartile range. To convert alanine aminotransferase, alkaline phosphatase, aspartate aminotransferase, creatinine kinase, and lactate dehydrogenase to μkat/L, multiply by 0.0167. BMI, body mass index; ICU, intensive care unit * Some patients presented more than one critically ill defining condition.

**Table 2:** Vital signs, radiological findings, and laboratory results in patients with Covid-19 on admission to ICU.

Vital Measurements (No. of Patients)	Value	Discharged Alive	Died	*p* Value
Total no.	156	69	87	
Temperature, °C (127)		59	68	
Median (IQR)	37.6 (37–38.4)	37.8 (37.2–38.4)	37.5 (37–38.3)	0.315
>38 °C	42 (33)	23	19	0.187
Oxygen Saturation (142)		67	75	
Median (IQR)	92 (87–95)	93 (88–95)	92 (85–95)	0.04
<90	53 (37.3)	22	31	0.296
Received Supplemental Oxygen (128)		58	70	0.561
Respiratory Rate (47)		23	24	
Median (IQR)	22 (16–30)	24 (18–32)	19 (12–29)	0.077
>20	25 (53.2)	14	11	0.302
Heart Rate, beats/min (139)		66	73	
Median (IQR)	91 (80–101)	91 (80–100)	91 (81–104)	0.331
<60	5 (3.6)	2	3	0.733
>100	36 (25.9)	15	21	0.804
Systolic Blood Pressure (139)		67	72	
Median (IQR)	135 (116–150)	132 (120–150)	136 (114–148)	0.791
Diastolic Blood Pressure (138)		66	72	
Median (IQR)	73 (60–82)	74 (65–83)	70 (60–82)	0.489
Chest X-ray (156)		69	87	
Normal (13)		4	9	0.302
Unilateral Filtration (21)		7	14	0.280
Bilateral Filtration (117)		57	60	0.051
Pleural Effusion (5)		1	4	0.268
**Initial laboratory measures, median (no.)**				
White Blood Cell Count, × 10^9^/L (149)		68	81	
Median (IQR)	8.29 (6–11.3)	7.75 (6–10.34)	8.6 (6–13)	0.048
<3.8 × 10^9^/L	11	6	5	0.538
>10.8 × 10^9^/L	41 (27.5)	14	27	0.083
Creatinine mg/dl (149)		68	81	
Median (IQR)	1 (0.8–1.4)	0.86 (0.66–1.06)	1.19 (0.8–1.67)	0.076
Aspartate aminotransferase, U/L (123)		62	61	
Median (IQR)	48 (30–75)	44 (28–72)	55 (31–88)	0.148
>40 U/L	71 (57.7)	34	37	0.514
Alanine aminotransferase, U/L (130)		65	65	
Median (IQR)	30 (19–54)	30 (21–62)	27 (18–52)	0.683
>60 U/L	26 (20)	17	9	0.079
C-Reactive Protein, mg/dL (141)		68	73	
Median (IQR)		20.16 (16–40.15)	23.7 (12–49.59)	0.959
D-dimer, ng/mL (80)				
Median (IQR)	1445 (887–2780)	1225 (780–2042)	1719 (1087–3622)	0.546

Abbreviations: Covid-19, coronavirus disease 2019; IQR, interquartile range. To convert alanine aminotransferase, alkaline phosphatase, aspartate aminotransferase, creatinine kinase, and lactate dehydrogenase to μkat/L, multiply by 0.0167.

**Table 3:** Summary of clinical measures and complications in critically ill Covid-19 patients treated in the ICU at study end point.

	No. (%)	Discharged Alive	Died	*p* Value
Total no.	156	69	87	
Interventions				
Oxygen	128	58	70	0.6
CPAP/BiPAP	39	29	10	<0.0001
Invasive Mechanical Ventilation	93	41	52	0.9
TPN	15	9	6	0.2
Enteral Nutrition	74	29	45	0.2
ECMO	6	5	1	<0.05
Renal Replacement Therapy	14	2	12	0.02
Tracheostomy	14	12	2	0.001
Medical Therapy				
Antibiotic Therapy	131	62	69	0.07
Anti-IL-6	9	6	3	0.15
Anti-Fibrinolytic Therapy	4	2	2	0.8
Glucocorticoid Therapy	34	20	14	
Inotropic Therapy	78	31	47	0.3
IVIG	7	5	2	0.1
Anti-Inflammatory	18	10	8	0.474
Antiviral Therapy	31	23	8	<0.001
Antimalarial Therapy	124	63	61	0.001
Complications				
Respiratory Failure	109	42	67	0.029
ARDS	59	25	34	0.716
Heart Failure	11	2	9	0.071
Sepsis	21	3	18	0.003
Septic Shock	29	9	20	0.113
Coagulopathy	4	3	1	0.209
Acute Cardiac Injury	8	3	5	0.694
Acute Kidney Injury	40	7	33	<0.0001
Secondary Infection	27	10	17	0.408

Antiviral therapy: remdesivir, lopinavir, and darunavir/cobicistat; antimalarial: hydroxychloroquine and chloroquine; anti-inflammatory: tocilizumab and interferon beta-1A.CPAP, Continuous positive airway pressure (non-invasive); BiPAP, Bilevel Positive Airway Pressure (non-invasive); TPN, Total parenteral nutrition; ECMO, Extracorporeal membrane oxygenation; ARDS, Acute respiratory distress syndrome.

**Table 4:** Univariable and multivariable analysis of outcome in critically ill Covid-19 patients in the ICU.

Variable	Univariable Analysis			Multivariable Analysis		
	OR	95% CI	*p* Value	OR	95% CI	*p* Value
Age, per year	1.11	1.07–1.15	<0.0001	1.08	1.03-1.13	0.0004
Sex			0.0048			0.183
Male	Reference			Reference		
Female	2.89	1.38–6.07		2.23	0.68–7.28	
HTN			<0.0001			0.532
Absent	Reference			Reference		
Present	4.96	250–9.81		1.45	0.45-4.66	
Diabetes			0.015			0.464
Absent	Reference			Reference		
Present	2.28	1.17–4.4		1.51	0.49–460	
IHD			0.030			0.614
Absent	Reference			Reference		
Present	2.53	1.09-5.90		1.36	0.40–4.57	
COPD			0.122			
Absent	Reference					
Present	2.85	0.75–10.81				
ARDS			0.259			
Absent	Reference					
Present	1.44	0.36–1.31				
Acute Organ Failure			0.227			
Absent	Reference					
Present	1.50	0.77-2.93				
Sepsis			0.002			0.013
Absent	Reference			Reference		
Present	3.69	1.61–8.43		4.97	1.40–17.69	
CRP (mg/dL), Per Unit	1.00	0.99–1.00	0.1985			
WBC (10^9^/L), Per Unit	1.07	0.99–1.15	0.033	1.05	0.93–1.17	0.392
Ventilation		0.596				
Absent	Reference					
Present	1.19	0.61–2.32				
Anti-Inflammatory *			0.074			
Not Administered	Reference					
Administered	0.52	0.25–1.06				
Antimalarial *			0.002			0.085
Not Administered	Reference			Reference		
Administered	0.22	0.08–0.58		0.30	0.08–1.17	
Antiviral *			0.0007			0.065
Not administered	Reference			Reference		
Administered	0.21	0.08–0.52		0.19	0.035–1.11	
Length of ICU Stay (days)	0.89	0.85–0.93	<0.001	0.92	0.84–0.96	0.0012

* Antiviral therapy: remdesivir, lopinavir, and darunavir/cobicistat; antimalarial: hydroxychloroquine and chloroquine; anti-inflammatory: tocilizumab and interferon beta-1a.HTN, hypertension; IHD, ischemic heart disease; COPD, chronic obstructive pulmonary disease; ARDS, acute respiratory distress syndrome; CRP, C reactive protein; WBC white blood cells.

## Supplementary Materials

The following are available online at https://www.mdpi.com/2077-0383/9/7/2282/s1. Figure S1: Covid-19 burden in Israel. A, Overall number of cases (*orange*) and deaths (*blue*). Logarithmic scale. B, Number of available ICU beds dedicated to SARS-CoV-2-positive patients (*orange*) and number of ICU beds occupied by critically ill Covid-19 patients.
