# Supplementary figures and images for: Clinical Course and Outcomes of Severe Covid-19: A National Scale Study
Source: J Clin Med. 2020 Jul 18;9(7):2282. doi: 10.3390/jcm9072282 (PMC7408944; doi:10.3390/jcm9072282)

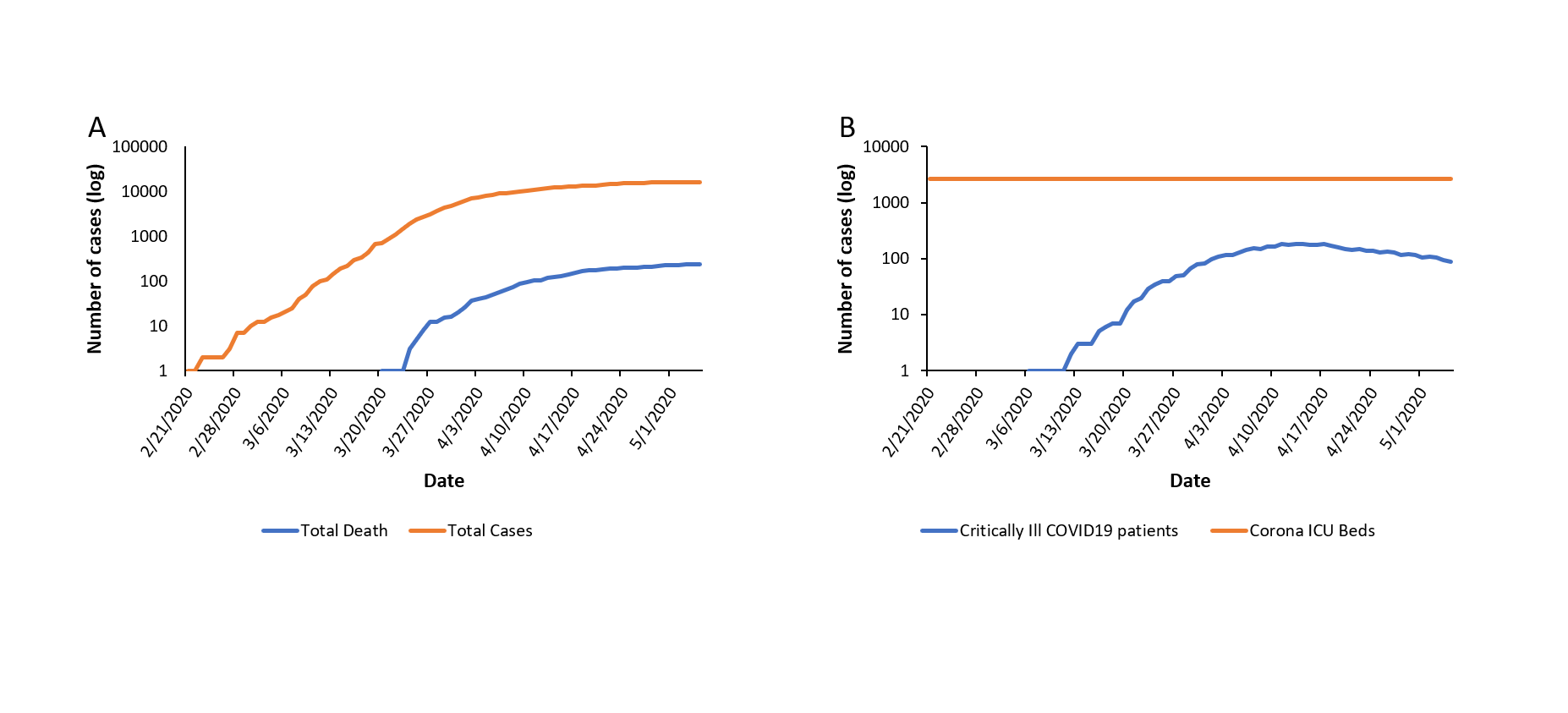

Supplement: Supplementary file 1 [file jcm-09-02282-s001.zip › jcm-848849-supplementary.tif]
